# Supplementary figures and images for: A novel selective PPARα modulator, pemafibrate promotes ischemia-induced revascularization through the eNOS-dependent mechanisms
Source: PLoS One. 2020 Jun 25;15(6):e0235362. doi: 10.1371/journal.pone.0235362 (PMC7316279; doi:10.1371/journal.pone.0235362)

# Supplemental Figure 1

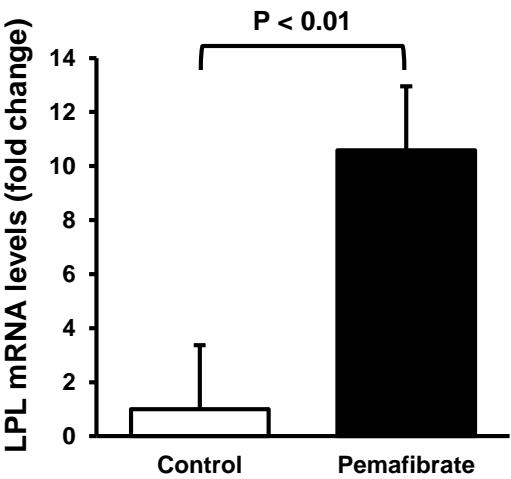

Supplement: S1 Fig — N = 3 in each group. (PDF) [file pone.0235362.s001.pdf]

# Supplemental Figure 2

A

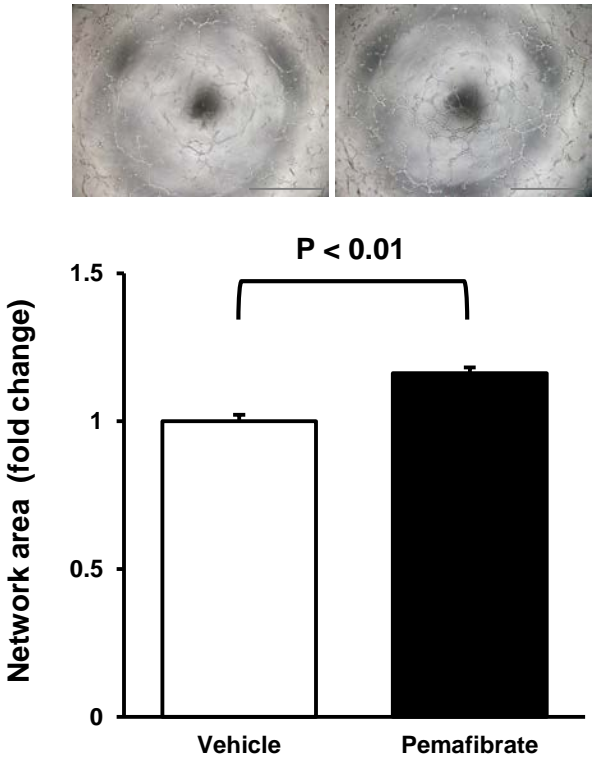

B

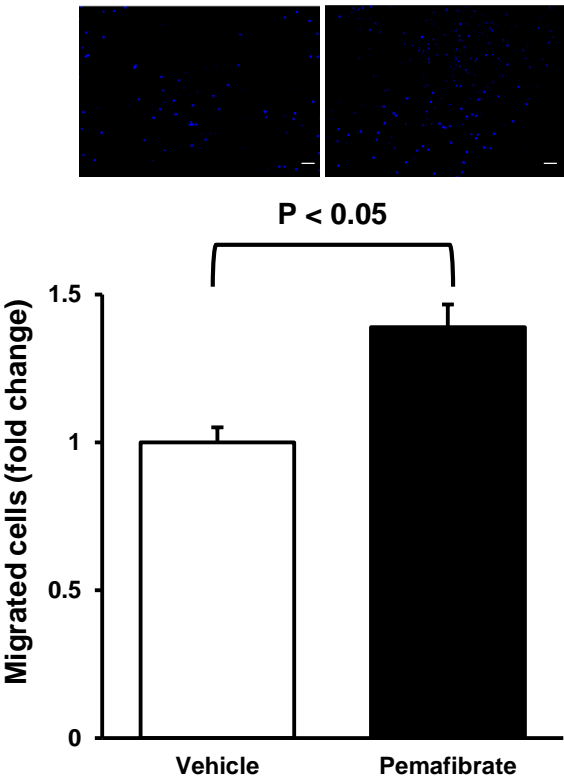

C

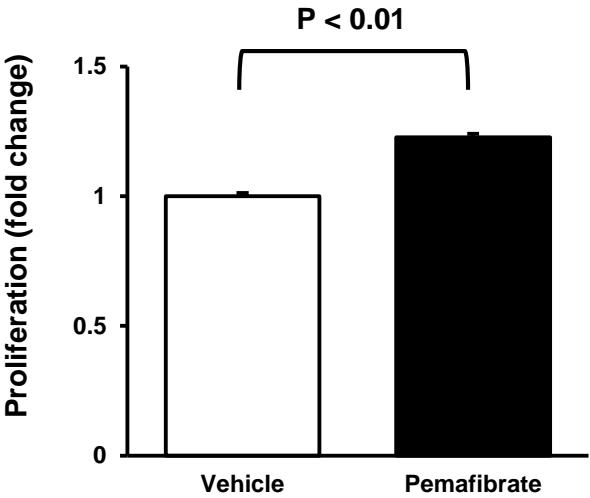

Supplement: S2 Fig — A. Endothelial cell network formation after treatment with pemafibrate under hypoxic condition. Upper panels show the representative photos of network formation of HUVECs at 8 h after treatment with pemafibate (10 nM) or vehicle. Lower panel shows the quantitative analysis of network area. N = 8 in each group. Scale bars show 1 mm. B. The number of migrated HUVECs at 8 h after treatment with pemafibrate (10 nM) or vehicle under hypoxic condition. Upper panels show the representative photos of DAPI staining of migrated HUVECs. N = 6 in each group. Scale bars show 200 μm. C. Proliferative activity of HUVECs at 8 h after treatment with pemafibrate (10 nM) or vehicle. N = 10 in each group. (PDF) [file pone.0235362.s002.pdf]

# Supplemental Figure 3

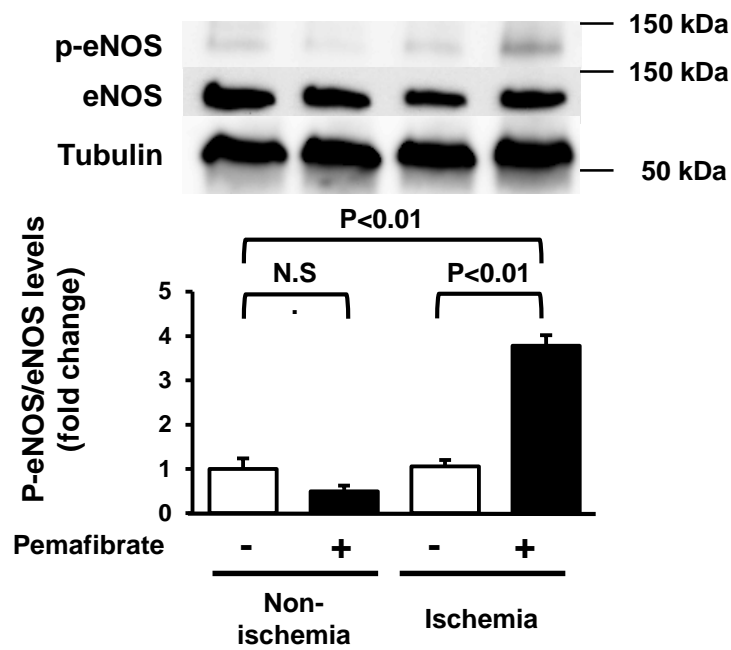

Supplement: S3 Fig — Upper panels show the representative blots of P-eNOS, eNOS and Tubulin at day 7 after surgery. Lower panel shows the quantitative analysis of phosphorylation levels of eNOS relative to eNOS. N = 4 in each group. (PDF) [file pone.0235362.s003.pdf]

# Supplemental Figure 4

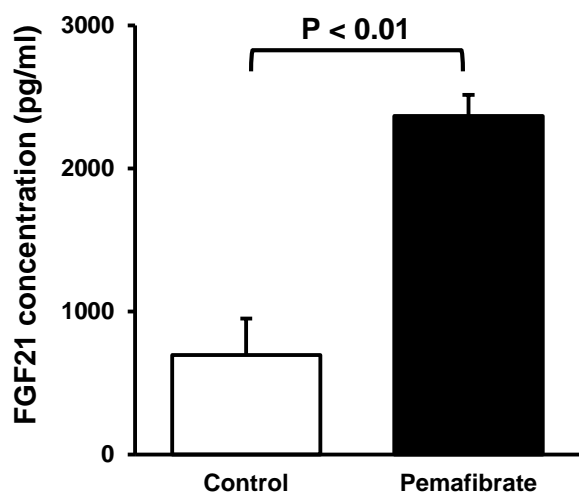

Supplement: S4 Fig — N = 5 in each group. (PDF) [file pone.0235362.s004.pdf]

# Supplemental Figure 5

## A Day 7

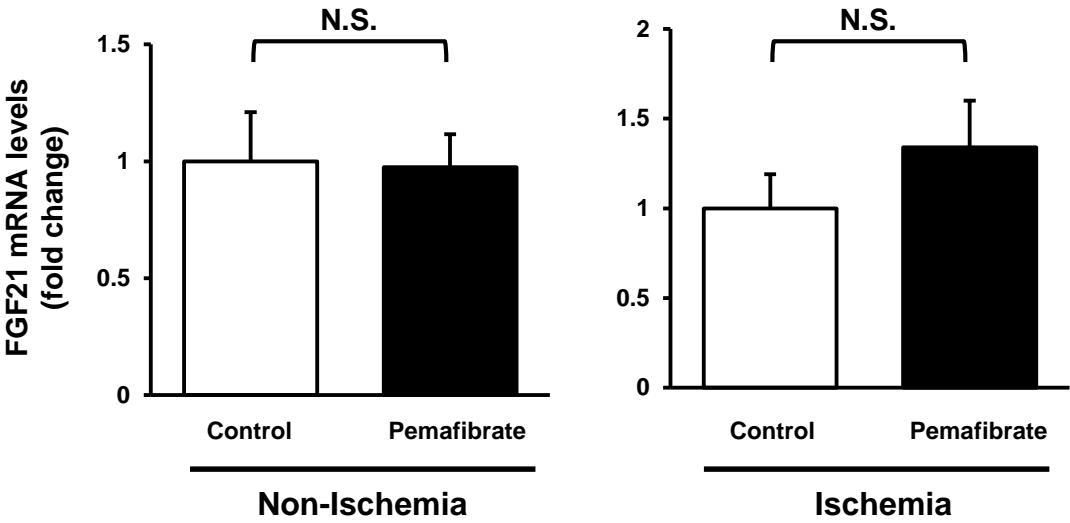

## B Day 28

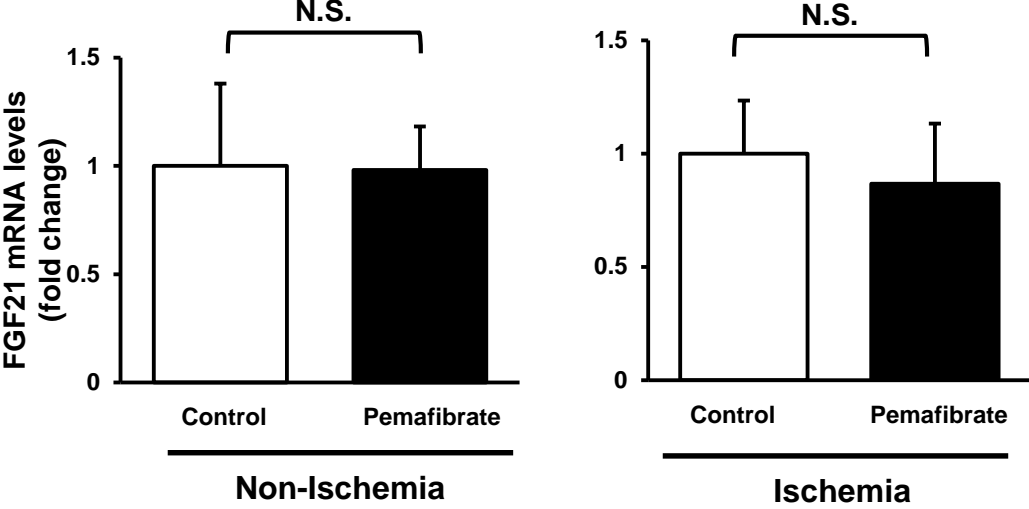

## C

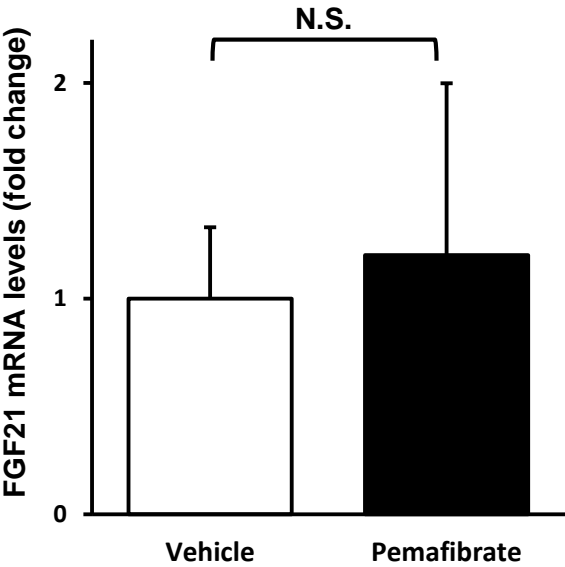

Supplement: S5 Fig — A and B. Pemafibrate did not affect the expression of FGF21 in non-ischemic and ischemic skeletal muscle at day 7 (A) and day 28 (B) after surgery. N = 8 in each group (A). N = 5 in each group (B). C. Treatment of HUVECs with pemafibrate had no effects on FGF21 expression. N = 5 in each group. (PDF) [file pone.0235362.s005.pdf]

# Supplemental Figure 6

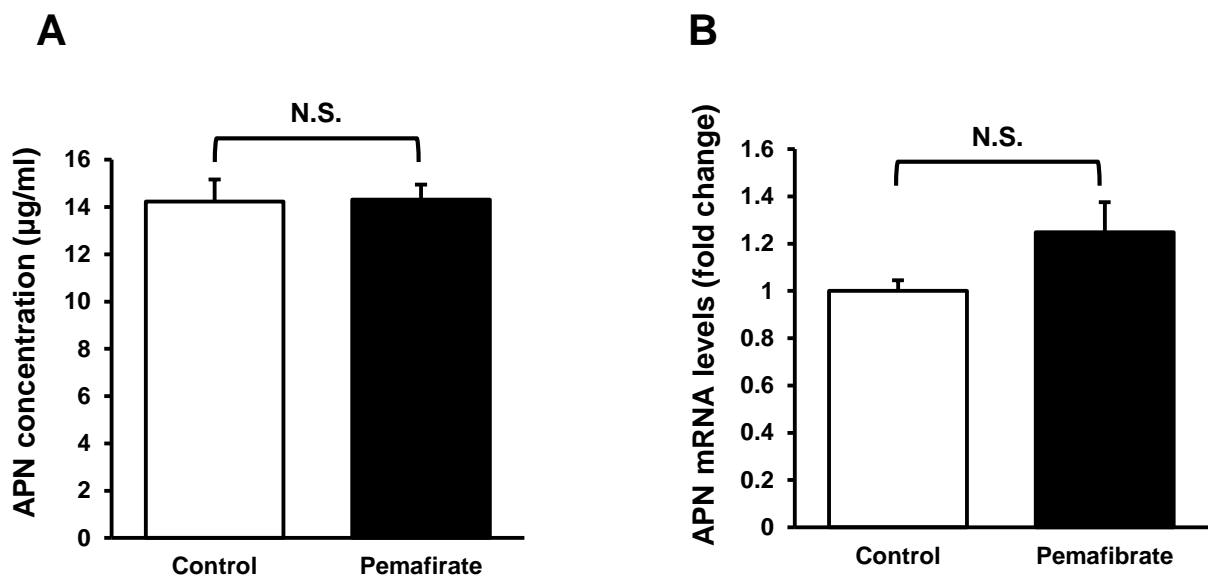

Supplement: S6 Fig — A. Plasma concentration of APN in WT mice fed control or pemafibrate diet. B. The mRNA expression of APN in epididymal fat tissue of WT mice fed control or pemafibrate diet. N = 8 in each group. (PDF) [file pone.0235362.s006.pdf]

# Supplemental Figure 7

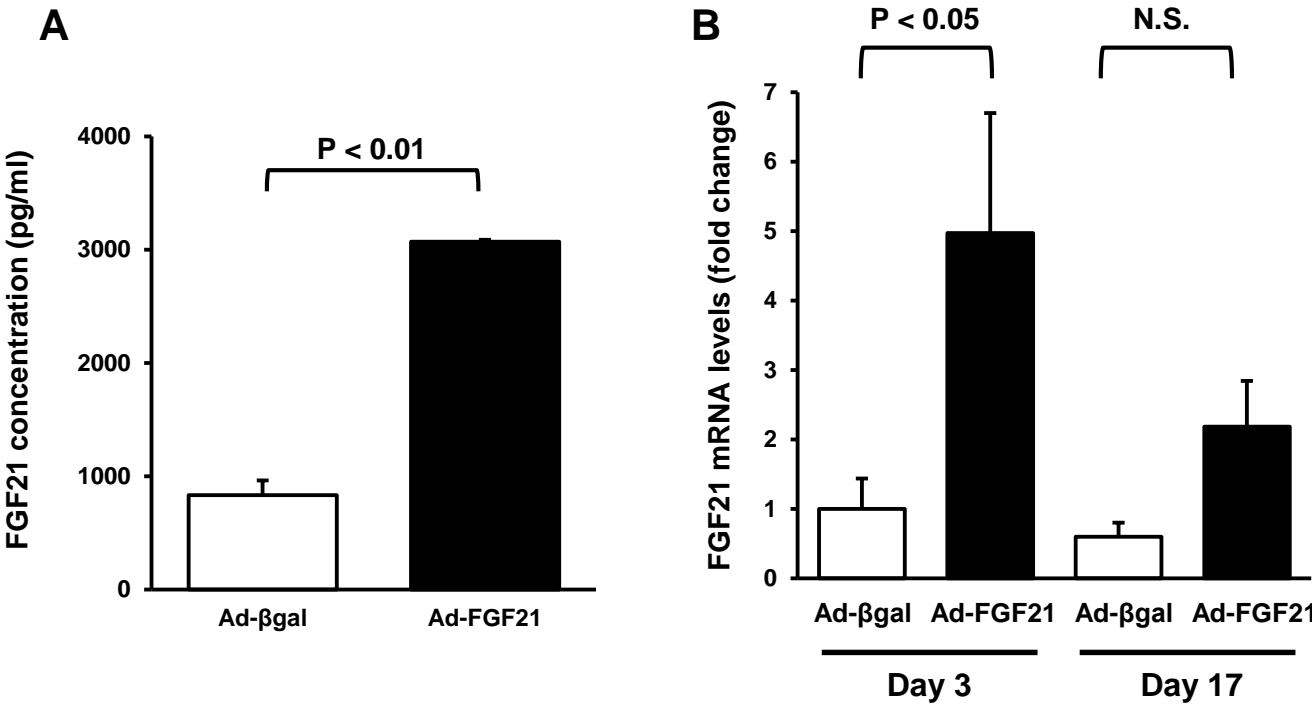

Supplement: S7 Fig — A. Plasma concentration of FGF21 in WT mice at day 17 after Ad-FGF21 or Ad-βgal administration as evaluated by ELISA system. N = 8 in each group. B. The mRNA levels of FGF21 in ischemic skeletal muscle at day 3 (N = 5 in each group) and day 17 (N = 8 in each group) after Ad-FGF21 or Ad-βgal administration. (PDF) [file pone.0235362.s007.pdf]

# Supplemental Figure 8

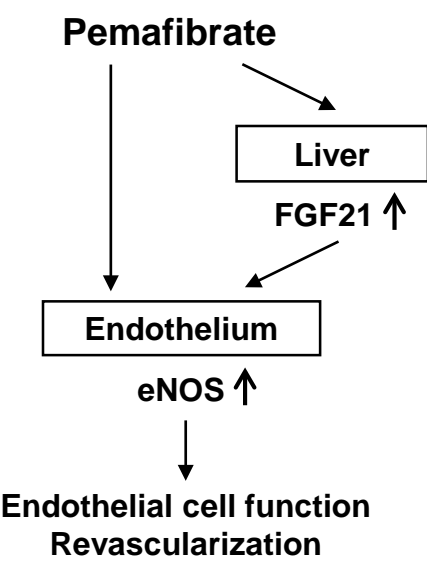

Supplement: S8 Fig — Pemafibrate directly activates eNOS signaling pathway in endothelium. Pemafibrate treatment leads to increases in hepatic FGF21 expression and circulating FGF21 levels, which in turn promote eNOS activation in endothelium. These two pathways are involved in regulation of endothelial cell function and revascularization. (PDF) [file pone.0235362.s008.pdf]

Figure 3A

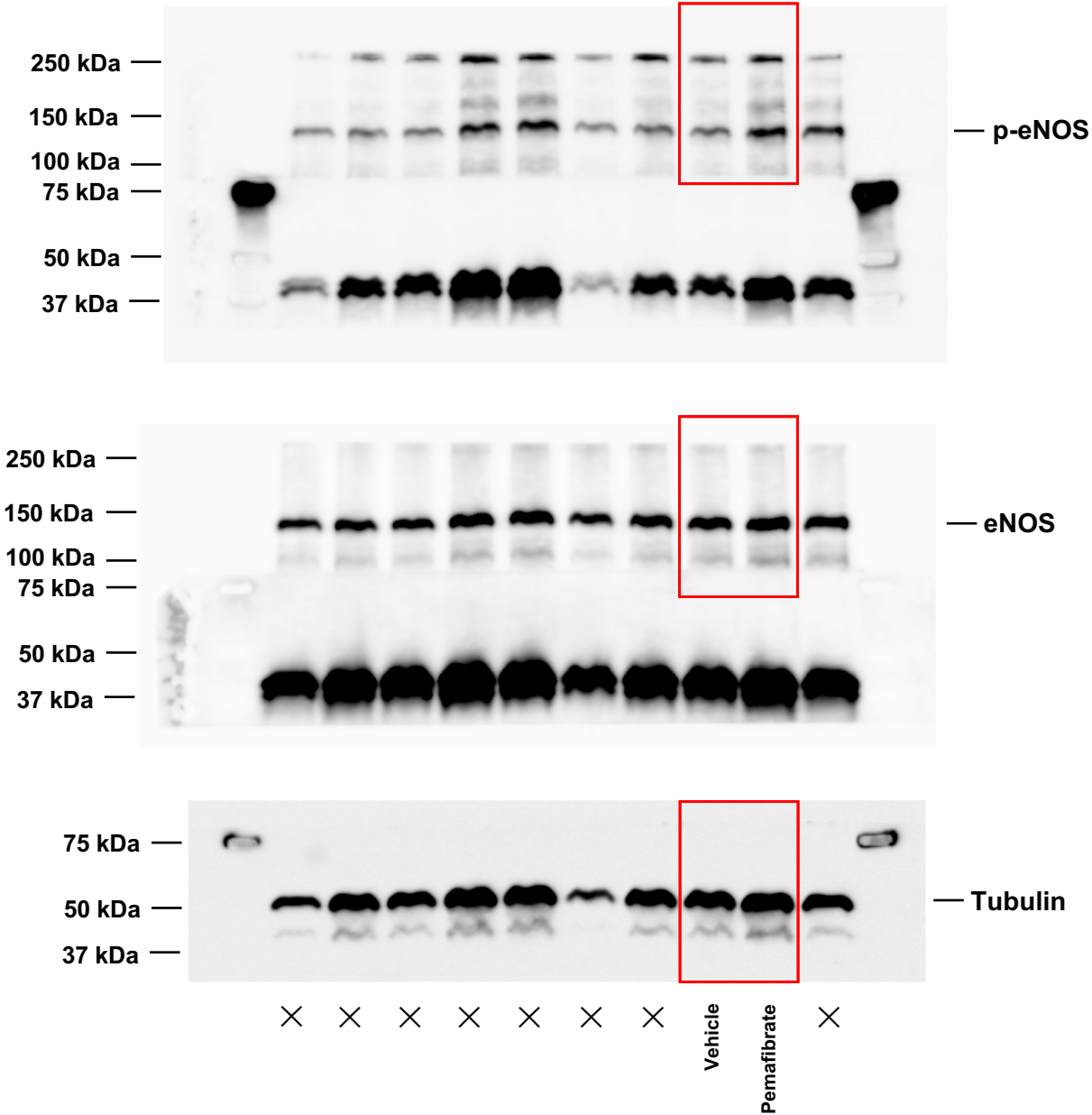

Figure 3D

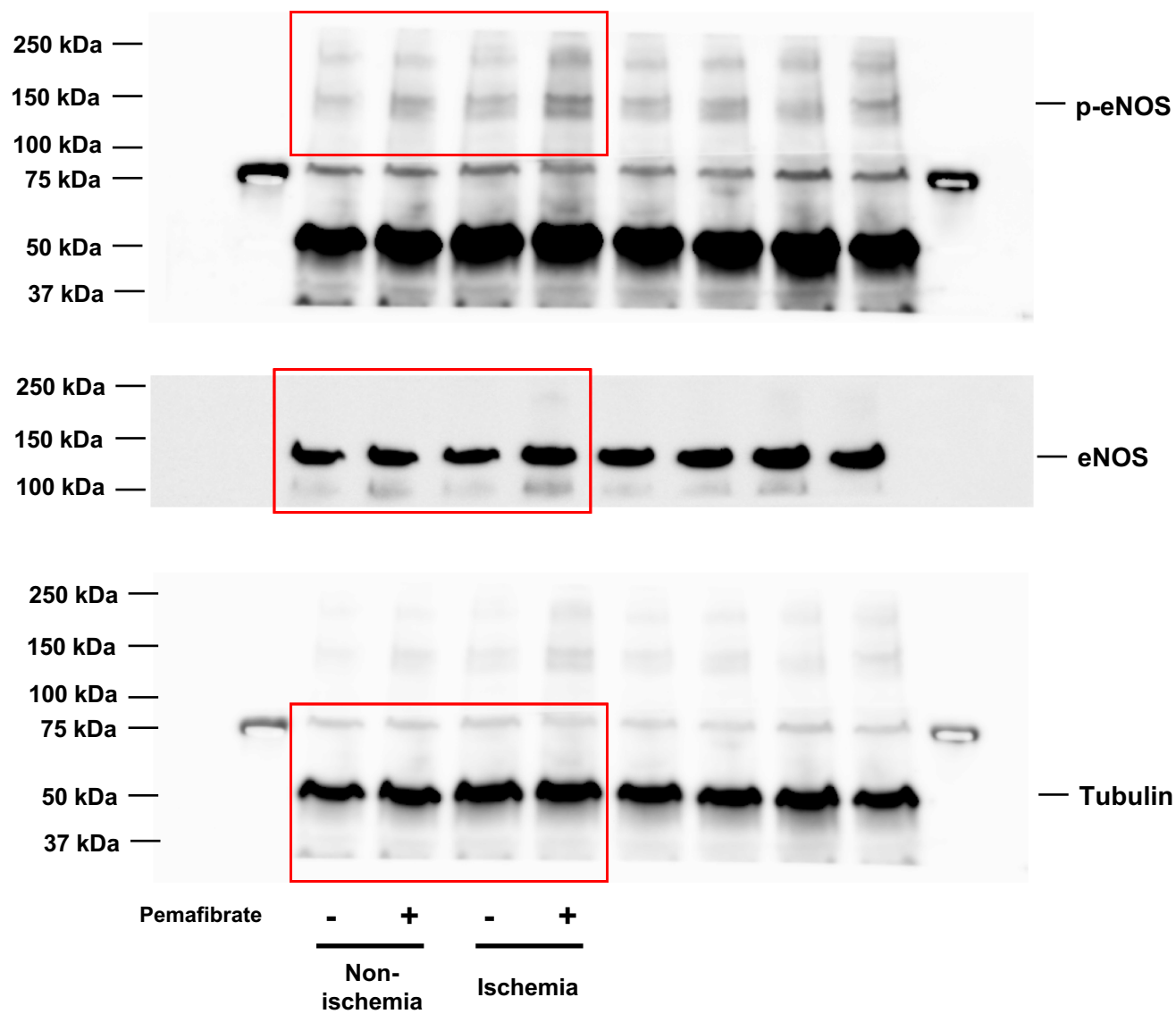

Figure 6A

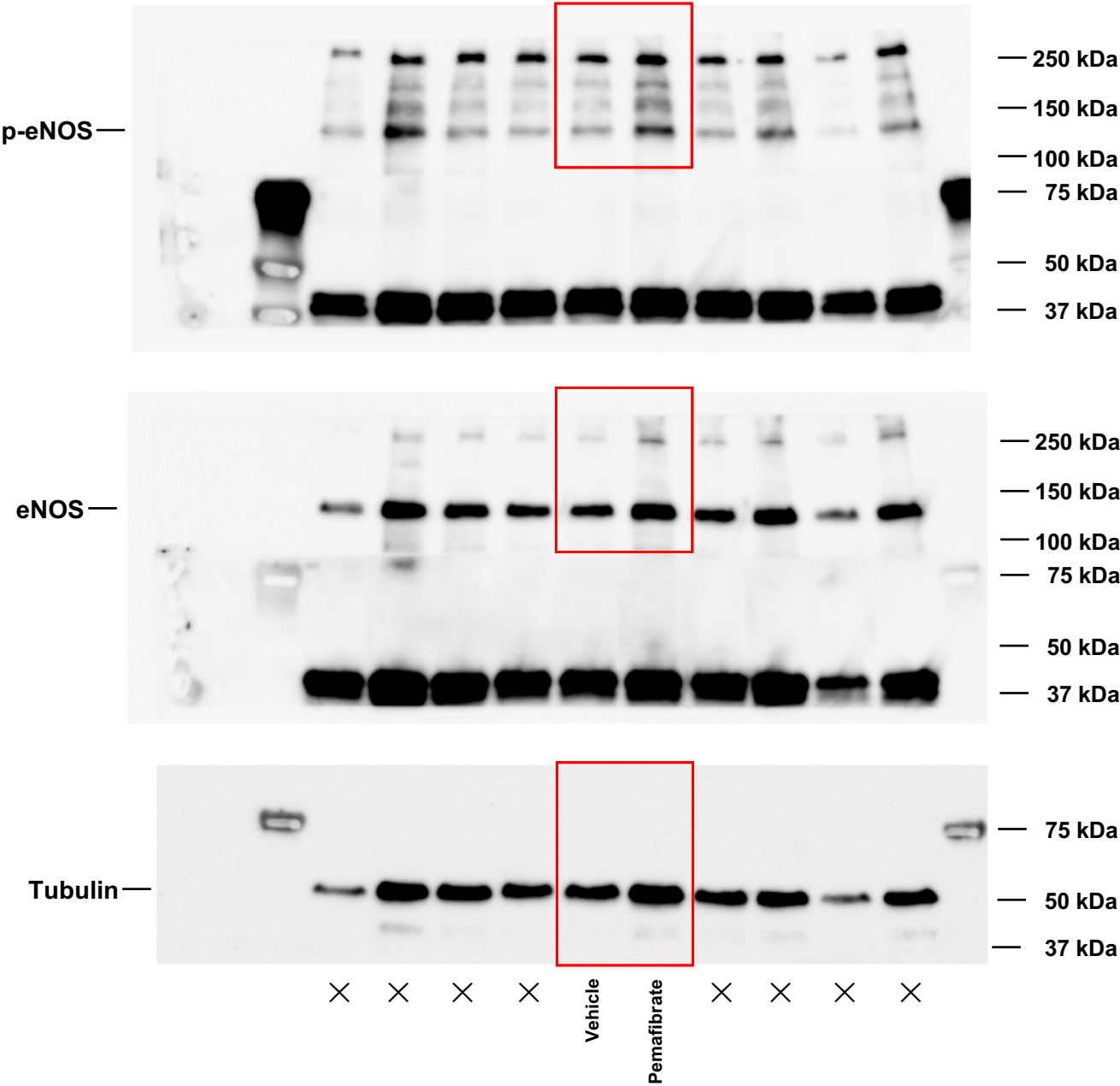

Figure 6E

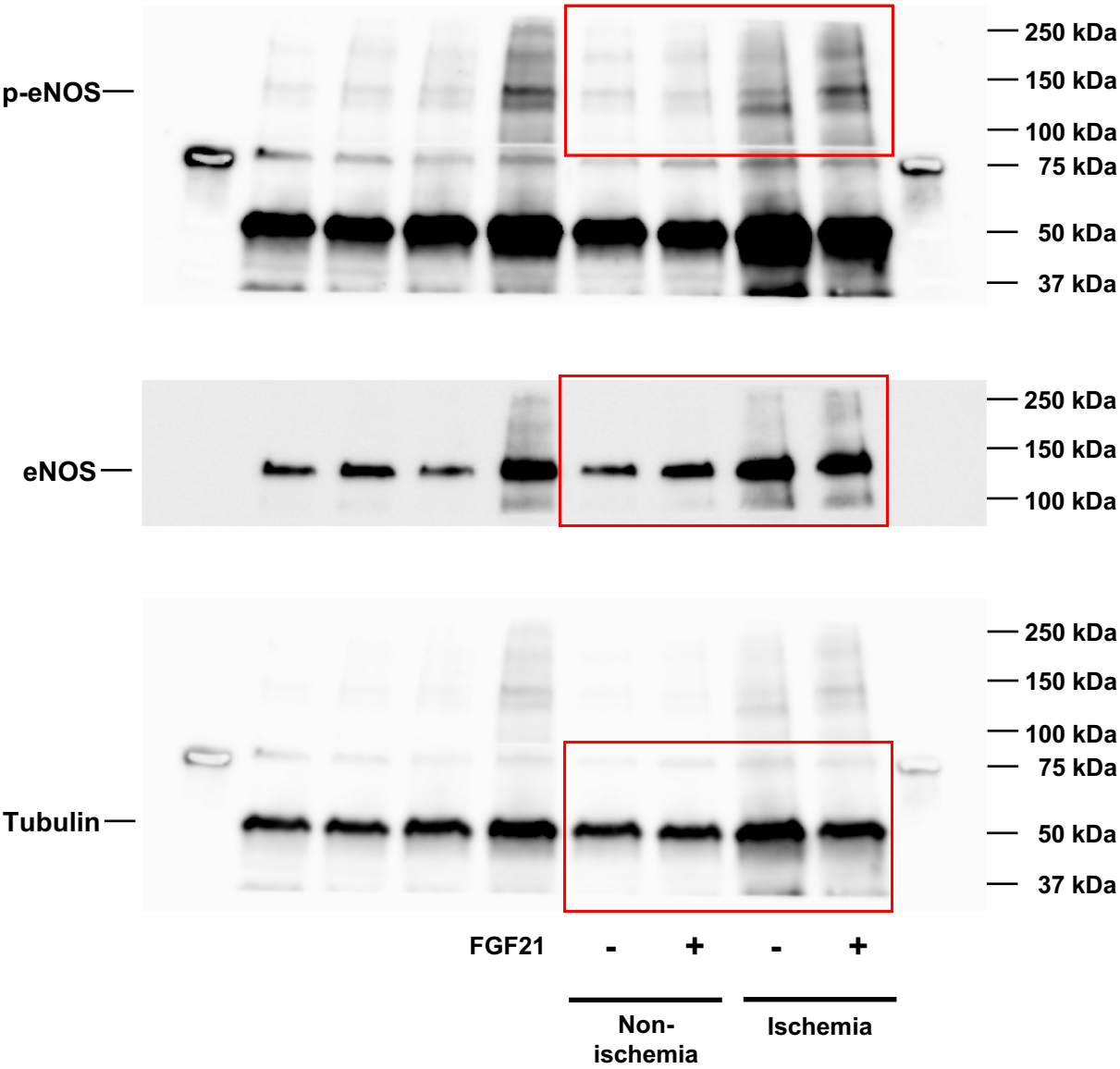

### Supplemental Figure 3

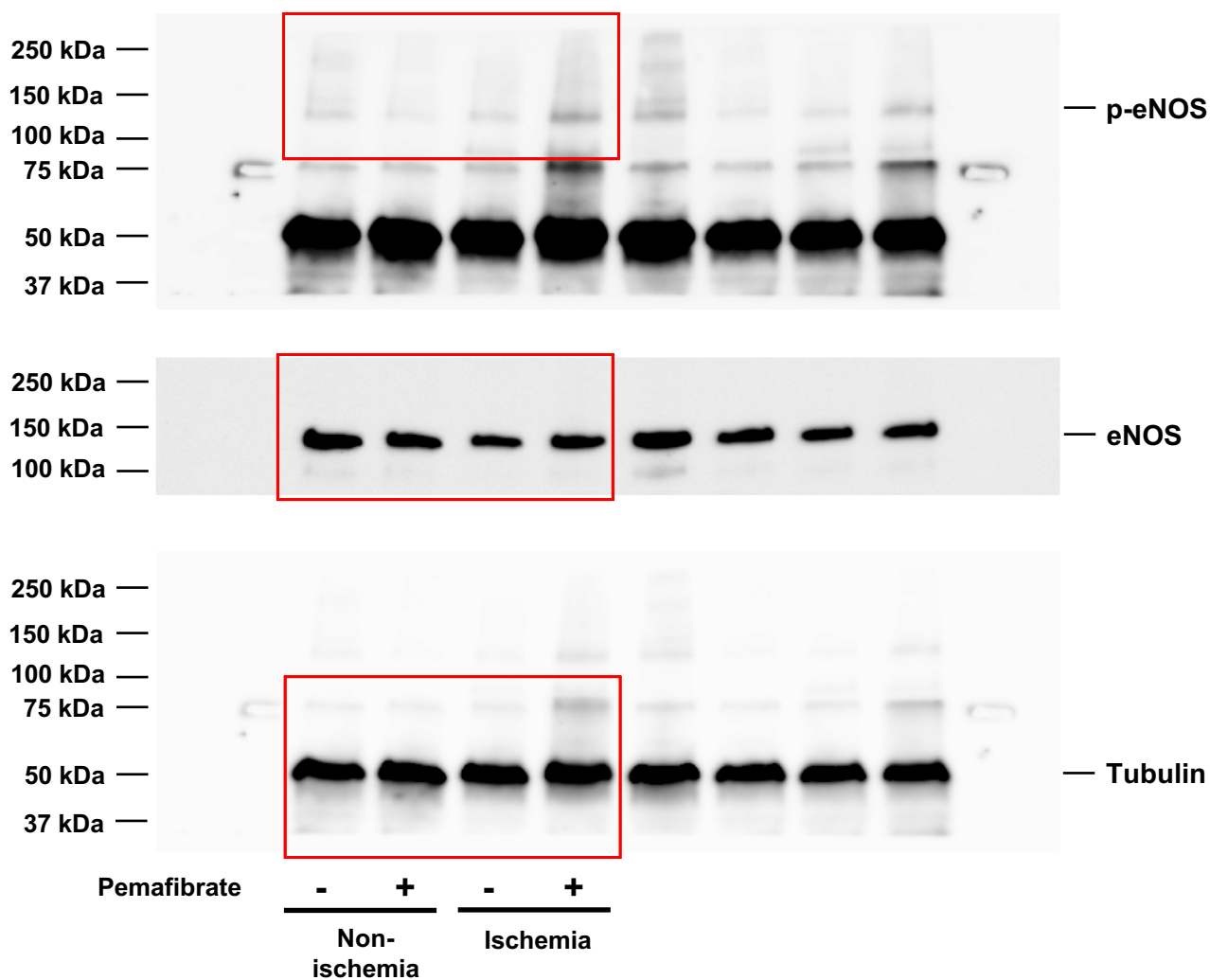

Supplement: S1 Raw Images — (PDF) [file pone.0235362.s009.pdf]
