# Supplementary material for: A novel selective PPARα modulator, pemafibrate promotes ischemia-induced revascularization through the eNOS-dependent mechanisms
Source: PLoS One. 2020 Jun 25;15(6):e0235362. doi: 10.1371/journal.pone.0235362 (PMC7316279; doi:10.1371/journal.pone.0235362)
Supplement: Attachment — Submitted filename: PONE-D-20-03337-review.docx [file pone.0235362.s010.docx]

**Review comments for PONE-D-20-03337**

The manuscript, a title “a novel selective PPARα modulator, pemafibrate promotes ischemia-induced revascularization through the eNOS-dependent mechanism” by Kawanishi H. et al is very interesting but need to present additional supporting data to prove the hypothesis.

***General comments.***

The main idea is that pemafibrate increase liver FGF21 and result in increasing circulated-FGF21 in blood which induces phosphorylation of eNOS to enhance ischemia-induced revascularization.

However, authors did not discuss why pemafibrate is effective only in ischemic tissue but not non-ischemic tissue. In addition, it is not clear even though authors suggested at least two mechanisms of pemafibrate at discussion. Please clarify how pemafibrate directly modulates endothelial behavior because authors only presented second effects of pemafibrate via FGF21 in current manuscript.

Authors presented in vitro effects of pemafibrate using HUVECs and showed that pemafibrate increases angiogenic characteristics such as cell migration, network-like tube formation, and proliferation. In vivo experiment, authors showed that pemafibrate was effective only in ischemic-hind limb tissue. It is not consistent between in vitro experiments and in vivo experiments because authors did all in vitro experiments under nomoxic condition.

In addition, authors used 28 days ischemic-limb tissues to show p-eNOS but 1h samples in HUVECs. It is highly possible that FGF21 increase p-eNOS early time point and then the p-eNOS plays a role to enhance revascularization pathway. It might be already finished revascularization at end point 28 days dependent on laser doppler images. Please show all time course for p-eNOS using hind limb ischemic tissues because it is important to check p-eNOS kinetics to enhance revascularization.

Please show ad-FGF21 expression levels in skeletal muscle tissues with time course.

Need to have more detail method section. For example, how authors measured plasma concentration of total choresterol, triglycerol, and glucose.

Please show p-eNOS levels in non-ischemic limb tissues with ischemic tissues in parallel.

Please clarify when authors measured plasma FGF21 concentration after administrating ad-FGF21.

The quantification of network area in Figure 2A is not matched with images. Please change with representative images.

Please show all images for tube formation and migration assays.

Please normalize p-eNOS levels by total eNOS levels instead of tubulin to clarify whether eNOS expression does not change by pemafibrate or FGF21.

Please show FGF21 levels in HUVEC treated with or without pemafibrate.

Please show plasma FGF21 levels with time course in non-ischemic and ischemic tissue.

Please clarify whether FGF21 is an initial key messenger of pemafibrate by showing rescue effect with FGF21 inhibitor or antibody.

Please show effects of pemafibrate in vitro under hypoxic condition to verify in vivo hind limb results.

The conclusion is overestimated because authors did not show any data for lipid metabolism.
