# Supplementary material for: A novel selective PPARα modulator, pemafibrate promotes ischemia-induced revascularization through the eNOS-dependent mechanisms
Source: PLoS One. 2020 Jun 25;15(6):e0235362. doi: 10.1371/journal.pone.0235362 (PMC7316279; doi:10.1371/journal.pone.0235362)
Supplement: Attachment — Submitted filename: Response to Reviewers.docx [file pone.0235362.s011.docx]

**Reviewer #1:**

**The manuscript, a title “a novel selective PPARα modulator, pemafibrate promotes ischemia-induced revascularization through the eNOS-dependent mechanism” by Kawanishi H. et al is very interesting but need to present additional supporting data to prove the hypothesis.**

**Response:** We thank the reviewer for these positive comments.

**General comments.**
**The main idea is that pemafibrate increase liver FGF21 and result in increasing circulated-FGF21 in blood which induces phosphorylation of eNOS to enhance ischemia-induced revascularization. However, authors did not discuss why pemafibrate is effective only in ischemic tissue but not non-ischemic tissue. In addition, it is not clear even though authors suggested at least two mechanisms of pemafibrate at discussion. Please clarify how pemafibrate directly modulates endothelial behavior because authors only presented second effects of pemafibrate via FGF21 in current manuscript.**

**Response:** We thank the reviewer for these suggestions. We examined the effects of pemafibrate on HUVEC behaviors in vitro under hypoxic condition. Network formation, migration and proliferation of HUVECs were promoted by pemafibrate treatment even under hypoxic condition. Thus, it is conceivable that pemafibrate treatment can enhance the revascularization and capillary density in ischemic limb of mice. These data are included in Supplemental Figure 2 and Results section (Page 9, line 16-18) in the revised manuscript.

We found that systemic delivery of pemafibrate or FGF21 enhanced eNOS phosphorylation in ischemic skeletal muscle tissues, but not in non-ischemic skeletal muscles. These findings are in agreement with the results that pemafibrate or FGF21 is effective at increasing capillary density only in ischemic tissue but not non-ischemic tissue. These data are included in Figure 3D, Supplemental Figure 3 , Results section (Page 9, line 26 – Page 10, line 3) and Discussion section (Page 13, line 2-6) in the revised manuscript.

In addition, we found that the stimulatory effects of pemafibrate on network formation and migration of HUVECs were abolished by GW6471, which is a specific inhibitor of PPARα, indicating that pemafibrate can directly modulate endothelial behavior in a PPARα dependent manner. These data are included in Figure 3B, 3C, Result section (Page 9, line 13-18) and Discussion section (Page 11, line 26 – Page 12, line 3) in the revised manuscript.

**Authors presented in vitro effects of pemafibrate using HUVECs and showed that pemafibrate increases angiogenic characteristics such as cell migration, network-like tube formation, and proliferation. In vivo experiment, authors showed that pemafibrate was effective only in ischemic-hind limb tissue. It is not consistent between in vitro experiments and in vivo experiments because authors did all in vitro experiments under normoxic condition.**

**Response:** We thank the reviewer for these important suggestions. We examined the effects of pemafibrate on HUVEC behaviors in vitro under hypoxic condition. Network formation, migration and proliferation of HUVECs were promoted by pemafibrate treatment even under hypoxic condition. Thus, it is conceivable that pemafibrate treatment can enhance the revascularization and capillary density in ischemic limb of mice. These data are included in Supplemental Figure 2 and Results section (Page 9, line 16-18) in the revised manuscript.

**In addition, authors used 28 days ischemic-limb tissues to show p-eNOS but 1h samples in HUVECs. It is highly possible that FGF21 increase p-eNOS early time point and then the p-eNOS plays a role to enhance revascularization pathway. It might be already finished revascularization at end point 28 days dependent on laser doppler images. Please show all time course for p-eNOS using hind limb ischemic tissues because it is important to check p-eNOS kinetics to enhance revascularization.**

**Response:** As the reviewer pointed out, p-eNOS could increase at earlier time point. Thus, we checked p-eNOS in skeletal muscles at day 7 after surgery. The levels of p-eNOS were higher in ischemic limb of pemafibrate-treated mice than in that of control mice at day 7. This result is now shown in Supplemental Figure 3 and Result section (Page 9, line 26 – Page 10, line 3) in the revised manuscript.

**Please show ad-FGF21 expression levels in skeletal muscle tissues with time course.**

**Response:** We thank the reviewer for the useful suggestion. We measured FGF21 expression of skeletal muscles both at day 3 and day 17 after adenoviral vector injection. The mRNA levels of FGF21 increased at skeletal muscle at 3 days after Ad-FGF21 injection compared with control Ad-βgal injection. The mRNA levels of FGF21 also tended to be higher at skeletal muscle at 17 days after Ad-FGF21 injection than Ad-βgal injection, but it was not statistically significant. These results are now included in Supplemental Figure 7B and Result section (Page 10, line 25 – Page 11, line 1).

**Need to have more detail method section. For example, how authors measured plasma concentration of total choresterol, triglycerol, and glucose.**

**Response:** We thank the reviewer for these suggestions. We prodived the more detailed description for measurement of total cholesterol, triglyceride and glucose in Materials and Methods sections (Page 5, line 15-16) in the revised manuscript.

**Please show p-eNOS levels in non-ischemic limb tissues with ischemic tissues in parallel.**

**Response:** According to the reviewer’s suggestion, we evaluated the p-eNOS levels in non-ischemic limb tissues, too. These results are now shown in Figure 3D and Supplemental Figure 3 in the revised manuscript.

**Please clarify when authors measured plasma FGF21 concentration after administrating ad-FGF21.**

**Response:** We measured plasma FGF21 concentration at day 3 after Ad-FGF21 administration. In addition, we evaluated plasma FGF21 concentration at day 17 after Ad-FGF21 administration. These data and points are included in Figure 4C, Supplemental Figure 7A and Result section (Page 10, line 22-25) in the revised manuscript.

**The quantification of network area in Figure 2A is not matched with images. Please change with representative images.**

**Response:** According to the reviewer’s suggestion, we added new representative photos of network formation in Figure 2A.

**Please show all images for tube formation and migration assays.**

**Response:** According to the reviewer’s suggestions, we added all images for tube formation and migration assay in the revised manuscript.

**Please normalize p-eNOS levels by total eNOS levels instead of tubulin to clarify whether eNOS expression does not change by pemafibrate or FGF21.**

**Response:** According to the reviewer’s suggestion, we normalized p-eNOS levels by total eNOS levels in Figure 3A, 3D, 6A, 6E and Supplemental Figure 3 in the revised manuscript.

**Please show FGF21 levels in HUVEC treated with or without pemafibrate.**

**Response:** We thank the reviewer for an important suggestion. We checked mRNA levels of FGF21 in the presence or absence of pemafibrate in HUVECs. Treatment with pemafibrate had no effect on the expression levels of FGF21 in HUVECs. These findings are now shown in Supplemental Figure 5C and Result section in our revised manuscript (Page 10, line 15-16).

**Please show plasma FGF21 levels with time course in non-ischemic and ischemic tissue.**

**Response:** We thank the reviewer for the suggestion. We measured plasma FGF21 levels at day 7 and day 28 after surgery. Treatment with pemafibrate increased plasma FGF21 levels both at day 7 and day 28 compared with control. These results are shown Supplemental Figure 4, Figure 4A and Result section in a revised manuscript (Page 10, line 9-11). In contrast, there were no difference in the mRNA levels of FGF21 in non-ischemic and ischemic skeletal muscle at day 7 and day 28 after surgery. These data are now shown in Supplemental Figure 5A, 5B and Result section in a revised manuscript (Page 10, line 11-15).

**Please clarify whether FGF21 is an initial key messenger of pemafibrate by showing rescue effect with FGF21 inhibitor or antibody.**

**Response:** It is important to clarify whether FGF21 is a key regulator of pemafibrate in angiogenic response in vivo. Thus, we should investigate the contribution of FGF21 to pemafibrate-stimulated revascularization by using FGF21 deficient mice or FGF21 inhibitors. The time limit to revise our manuscript was May 2, which was around 6 weeks later after the first decision. It takes more than 6 weeks to perform these experiments. In addition, we believe that this is beyond the scope of this paper. Thus, future studies using FGF21 deficient mice or FGF21 inhibitors will be required to clarify whether FGF21 is essential for the pro-angiogenic effects of pemafibrate. These points are included in the discussion section in the revised manuscript (Page 12, line 19-21).

**Please show effects of pemafibrate in vitro under hypoxic condition to verify in vivo hind limb results.**

**Response:** We thank the reviewer for these suggestions. We examined the effects of pemafibrate on HUVEC behaviors in vitro under hypoxic condition. Network formation, migration and proliferation of HUVECs were promoted by pemafibrate treatment even under hypoxic condition. These findings are now included in Supplemental Figure 2, Result section (Page 9, line 16-18) and Methods section (Page 7, line 22-25) in the revised manuscript.

**The conclusion is overestimated because authors did not show any data for lipid metabolism.**

**Response:** We agree with the reviewer. We deleted the sentence “Clinically, pemafibrate treatment can ameliorate the atherogenic lipid profiles.” We also changed from the phrase “cardiovascular disease” to “peripheral arterial disease” in Discussion section (Page 14, line 9-10). In addition, we deleted the phrase “among patients with dyslipidemia” in conclusion in the abstract.

**Reviewer #2:**

**The manuscript entitled "A novel selective PPARα modulator, pemafibrate promotes ischemia-induced revascularization through the eNOS-dependent mechanisms" is well written. However, the authors need to clarify the following concerns.**

**1. Throughout the manuscript the authors have referred the figure 6 as figure 5 which is confusing and need serious attention.**

**Response:** We thank the reviewer for carefully reading our manuscript. We corrected our mistake in the revised manuscript.

**2. The IF staining of CD31 in muscle is not much informative. It is very difficult to understand ischemic region without nuclear staining. IHC staining of the same will be more acceptable.**

**Response:** We thank the reviewer for an important suggestion. We added photos of nuclear staining (DAPI) with CD31 staining in Figure 1C and 4E in the revised manuscript.

**3. The quality of blots are not good enough, such as in figure 3D, 6E.**

**Response:** We thank the reviewer for these suggestions. The quality of blots has been improved in Figure 3D and 6E in the revised manuscript.

**4. The bar graph for eNOS phosphorylation should be expressed in term of p-eNOS/total eNOS.**

**Response:** According to the reviewer’s suggestion, we evaluated p-eNOS levels in p-eNOS/total eNOS in Figure 3A, 3D, 6A, 6E and Supplemental Figure 3 in the revised manuscript.

**5. According to authors, pemafibrate stimulates revascularization through direct effect on endothelial cells. But no mechanism has been provided or predicted. Pemafibrate stimulated revascularization through increased expression of FGF21 does not explain the in-vitro effect.
6. The effect of pemafibrate on FGF21 is well known and the effect of FGF21 on endothelial cell proliferation through eNOS has also been reported. Therefore, the significance of the manuscript can be enhanced if the authors could find the mechanism of direct effect of pemafibrate on ECs.**

**Response:** We thank the reviewer for these important suggestions. we found that the stimulatory effects of pemafibrate on network formation and migration of HUVECs were abolished by GW6471, which is a specific inhibitor of PPARα, indicating that pemafibrate can directly modulate endothelial behavior in a PPARα dependent manner. These data are included in Figure 3B, 3C and Result section (Page 9, line 13-16) in the revised manuscript.
