# Supplementary material for: A novel selective PPARα modulator, pemafibrate promotes ischemia-induced revascularization through the eNOS-dependent mechanisms
Source: PLoS One. 2020 Jun 25;15(6):e0235362. doi: 10.1371/journal.pone.0235362 (PMC7316279; doi:10.1371/journal.pone.0235362)
Supplement: Attachment — Submitted filename: PONE-D-20-03337-R1 review.docx [file pone.0235362.s012.docx]

**PONE-D-20-03337R1**

Authors addressed almost reviewer’s comments and all responses are reasonable.

However, reviewer would like to request minor revision as following.

1. It is very important to reproduce authors’ research. Therefore, please provide exact information (company and catalog number, if you modified procedures, please describe it) of experimental assay kits.
2. In the same line, please clarify hypoxic condition (oxygen concentration) and provide a positive control to show that experimental system works well.
3. All images should have scale bars.
4. Almost images are very small. Please provide enlarged images with scale bars.
5. Western blotting images also should have molecular markers.
6. Please carefully check authors’ response. Because authors have wrong response about a part of first comment. Reviewer guess authors just copied and pasted it from response about second comment.
