# Supplementary material for: A novel selective PPARα modulator, pemafibrate promotes ischemia-induced revascularization through the eNOS-dependent mechanisms
Source: PLoS One. 2020 Jun 25;15(6):e0235362. doi: 10.1371/journal.pone.0235362 (PMC7316279; doi:10.1371/journal.pone.0235362)
Supplement: Attachment — Submitted filename: Response to Reviewers 060220.doc [file pone.0235362.s013.doc]

**Response to Reviewer 1**

**Authors addressed almost reviewer’s comments and all responses are reasonable.
However, reviewer would like to request minor revision as following.**

**Response:** We thank the reviewer for the positive comment.

**1. It is very important to reproduce authors’ research. Therefore, please provide exact information (company and catalog number, if you modified procedures, please describe it) of experimental assay kits.**

**Response:** We thank the reviewer for the important suggestion. We provided vendor names, catalog numbers of experimental assay kits in our revised manuscript.

**2. In the same line, please clarify hypoxic condition (oxygen concentration) and provide a positive control to show that experimental system works well.**

**Response:** We thank the reviewer for the important suggestion. HUVECs were exposed to 5% oxygen concentration by using Anaeropack system. We previously exposed cultured cardiomyocytes (rat neonatal ventricular myocytes) and HUVECs to hypoxia using this system ^1-5^. Cardiomyocytes led to more than 20% apoptosis after 12 h hypoxia following 24 h normoxia stimulation in this system. We described the information of oxygen concentration and carbon dioxide concentration in the revised manuscript (Page 8, line 4).

**3. All images should have scale bars.**

**Response:** We thank the reviewer for the important suggestion. All photos have scale bars in our revised Figure and Supplemental Figure.

**4. Almost images are very small. Please provide enlarged images with scale bars.**

**Response:** We thank the reviewer for the important suggestion. We enlarged photos in the revised Figures.

**5. Western blotting images also should have molecular markers.**

**Response:** We thank the reviewer for the important suggestion. We showed molecular weight markers of Western blots in our revised Figures and Supplemental Figures.

**6. Please carefully check authors’ response. Because authors have wrong response about a part of first comment. Reviewer guess authors just copied and pasted it from response about second comment.**

**Response:** We regret our wrong response in first comment. The reviewer suggested discussion of the reason why pemafibrate promotes angiogenic response only under ischemic condition. Thus, we discussed this point in Discussion section (Page 13, line 14 -17).

**References**

1.	Ogura Y, Ouchi N, Ohashi K, Shibata R, Kataoka Y, Kambara T, Kito T, Maruyama S, Yuasa D, Matsuo K, Enomoto T, Uemura Y, Miyabe M, Ishii M, Yamamoto T, Shimizu Y, Walsh K, Murohara T. Therapeutic impact of follistatin-like 1 on myocardial ischemic injury in preclinical animal models. *Circulation*. 2012;126:1728-1738

2.	Kataoka Y, Shibata R, Ohashi K, Kambara T, Enomoto T, Uemura Y, Ogura Y, Yuasa D, Matsuo K, Nagata T, Oba T, Yasukawa H, Numaguchi Y, Sone T, Murohara T, Ouchi N. Omentin prevents myocardial ischemic injury through amp-activated protein kinase- and akt-dependent mechanisms. *J Am Coll Cardiol*. 2014;63:2722-2733

3.	Yuasa D, Ohashi K, Shibata R, Mizutani N, Kataoka Y, Kambara T, Uemura Y, Matsuo K, Kanemura N, Hayakawa S, Hiramatsu-Ito M, Ito M, Ogawa H, Murate T, Murohara T, Ouchi N. C1q/tnf-related protein-1 functions to protect against acute ischemic injury in the heart. *FASEB J*. 2016;30:1065-1075

4.	Ohashi K, Enomoto T, Joki Y, Shibata R, Ogura Y, Kataoka Y, Shimizu Y, Kambara T, Uemura Y, Yuasa D, Matsuo K, Hayakawa S, Hiramatsu-Ito M, Murohara T, Ouchi N. Neuron-derived neurotrophic factor functions as a novel modulator that enhances endothelial cell function and revascularization processes. *J Biol Chem*. 2014;289:14132-14144

5.	Joki Y, Ohashi K, Yuasa D, Shibata R, Kataoka Y, Kambara T, Uemura Y, Matsuo K, Hayakawa S, Hiramatsu-Ito M, Kanemura N, Ito M, Ogawa H, Daida H, Murohara T, Ouchi N. Neuron-derived neurotrophic factor ameliorates adverse cardiac remodeling after experimental myocardial infarction. *Circ Heart Fail*. 2015;8:342-351

**Response to Reviewer #2**

**Although the authors have tried to answer the queries raised in previous review, still some questions have remain unanswered or not satisfactory.**

**Response:

1. CD31 staining in ischemic and non ischemic muscle is still not convincing. There are no bar graphs in the figures.**

**Response:** We thank the reviewer for the important comment. We showed the quantitative graphs on the right side of representative CD31 staining.

**2. Since, pemafibrate stimulated EC function in both normoxic and hypoxic condition in vitro, it fails to explain the effect of pemafibrate on ischemic tissue only.**

**Response:** The reviewer raised a good point. The reason for the discrepancy between in vivo and in vitro effects of pemafibrate on angiogenic response is unknown. This requires future investigation. These points are included in the discussion section in the revised manuscript (Page 13. Line 14-17).

**3. Treatment of EC with pemafibrate and GW6471 together could not explain the direct effect of pemafibrate on EC function independent of FGF21 since FGF21 is downstream to PPARα.**

**Response:** We thank the reviewer for these important suggestions. The stimulatory effects of pemafibrate on endothelial cell function were abolished by PPARα inhibition, indicating that pemafibrate can modulate endothelial behavior in a PPARα dependent manner. Importantly, although FGF21 is a target gene of PPARα, pemafibrate did not affect FGF21 expression in cultured endothelial cells. Thus, it is likely that pemafibrate can affect endothelial cell  function in a FGF21 independent manner. These data suggest that pemafibrate can directly modulate endothelial behavior via a PPARα signaling mechanism that is independent of FGF21 induction.  These points are included in the discussion section in the revised manuscript (Page 12, line 5 - 12).
